# Patients With Very Mild Dementia May Confuse Objective Cognitive Impairments With Subjective Physical Health of Quality of Life: The Tome City Project in Japan

**DOI:** 10.3389/fpsyg.2018.00533

**Published:** 2018-04-12

**Authors:** Mari Kasai, Kenichi Meguro

**Affiliations:** Division of Geriatric Behavioral Neurology, Cyclotron and Radioisotope Center, Tohoku University, Sendai, Japan

**Keywords:** elderly, dementia, quality of life, cognitive impairment, WHOQOL-BREF

## Abstract

Many elderly people with cognitive dysfunction may observe a decrease in their health levels and quality of life (QOL). The basic concept of QOL consists of several categories including physical functions and mental health. The QOL domain that is most important for elderly people is physical health and, to a lesser extent, psychological health, social relationships, and/ or the environment. Our aim was to explore the relationships between the subjective measure of QOL, an abbreviated version of the World Health Organization Quality of Life (WHOQOL-BREF) scale, and the objective measure of impairment, Clinical Dementia Rating (CDR), among elderly people in a community. Totally, 178 community dwellers aged 75 years and above agreed to participate and completed the WHOQOL-BREF; 66 (32 males, 34 females) scored a CDR of 0 (healthy), 86 (33, 53) scored a CDR of 0.5 (questionable dementia or very mild dementia), and 26 (12, 14) scored a CDR of 1 and above (dementia). According to Pearson’s correlation coefficient analysis (significance level, *p* < 0.05), the physical domain of the WHOQOL-BREF had significant statistical negative correlations with all CDR subscales. The CDR subscale of memory impairment had a significant statistical negative correlation with the WHOQOL-BREF subscales of the physical (*r* = -0.151, *p* = 0.044) and psychological (*r* = -0.232, *p* < 0.002) domains. The CDR subscale of home and hobbies impairment had significant statistical negative correlations with all WHOQOL-BREF subscales including the physical (*r* = -0.226, *p* = 0.002), psychological (*r* = -0.226, *p* = 0.002), social (*r* = -0.167, *p* = 0.026), and environmental (*r* = -0.204, *p* = 0.006) domains. Patients with very mild dementia may confuse cognitive impairment and physical disabilities. In the future, we need to systematically combine memory clinics and all departments related to the elderly for the successful early detection and rehabilitation of, and long-term care for, dementia.

## Introduction

Many elderly people who have cognitive dysfunction may notice a decrease in their health levels and quality of life (QOL). The World Health Organization (WHO) (2017a) defines health as “a state of complete physical, mental, and social well-being and not merely the absence of disease or infirmity.” In addition, it defines QOL as “an individual’s perception of their position in life in the context of the culture and value systems in which they live and in relation to their goals, expectations, standards and concerns” (The World Health Organization [WHO], 2017b). The basic concept of QOL consists of several categories including physical functions and mental health. However, actual health-related QOL among the elderly is a complex problem since most elderly people have several complicated chronic diseases and suffer from decreased physical functions as a result of aging.

In elderly people, cognitive impairments may affect their poor QOL in everyday life. We need to study the relationship between cognitive impairments and QOL in the daily activities of the elderly. To assess the severity of clinical dementia in the daily activities of the elderly, the Clinical Dementia Rating (CDR) scale is widely used. It is a semi-structured interview scale with six domains and five impairment levels (Hughes et al., 1982; Morris, 1993). The CDR scoring includes an interview by a physician and a clinical examination, as well as observations by family members. In this study, public health nurses visited subjects’ homes to evaluate their daily activities. A CDR of 0.5 (Hughes et al., 1982; Morris, 1993) or mild cognitive impairment (MCI) (Petersen et al., 1999; Petersen, 2004) is the boundary and transitional state between normality and dementia. A CDR of 0.5 is comparable to questionable dementia (Hughes et al., 1982; Morris, 1993). People with a CDR of 0.5 have some neuropsychological impairments, such as loss of memory, decline in executive function (Hashimoto et al., 2004; Nakata et al., 2009; Kasai et al., 2012), and apathy (Nakamura et al., 2013). In older adults with very mild Alzheimer’s disease, subjective complaints of dementia are not always amnesia. According to previous studies, older adults with MCI expressed subjective complaints of disturbed social activity (Meguro et al., 2004). Another objective observation study’s findings revealed impaired instrumental activities of daily living (IADL) in females (Ouchi et al., 2012, 2016). Such disturbed social activities were suspected of being associated with impaired QOL.

The abbreviated version of the World Health Organization Quality of Life (WHOQOL-BREF) scale is a brief questionnaire used to assess international cross-culturally-comparable QOL among young and elderly people, consisting of physical health, psychological health, social relationships, and environment domains (The WHOQOL Group, 1998; Saxena et al., 2001; Tazaki and Nakane, 2007). Lucas-Carrasco et al. (2011) reported that the WHOQOL-BREF had good reliability and validity for dementia. In a previous study of QOL in subjects with MCI, using the WHOQOL-BREF, Muangpaisan et al. (2008) reported that MCI patients had significantly lower psychological QOL than normal subjects. The QOL domain that is most important for elderly people is physical health followed by, to a lesser extent, psychological health, social relationships, and/or the environment.

Both CDR and WHOQOL-BREF are standardized scales to assess the everyday life of elderly people with dementia (Hughes et al., 1982; Morris, 1993; Lucas-Carrasco et al., 2011). CDR is a scale of objective cognitive impairments such as memory and home and hobbies impairments in daily living (Hughes et al., 1982; Morris, 1993), while the WHOQOL-BREF is an assessment of subjective QOL such as the physical and psychological domains of everyday life (The WHOQOL Group, 1998; Saxena et al., 2001; Tazaki and Nakane, 2007).

However, the relationship between objective cognitive impairments and subjective QOL, which includes physical, psychological, social, and environmental domains, has so far not been investigated in cases of very mild dementia. Our aim was to explore the relationships between the subjective measure (WHOQOL-BREF) and the objective measure (CDR) among elderly people in a community to contribute to the early detection of dementia and to identify the most important QOL domain among elderly people in Japan.

## Materials and Methods

### Participants

From November 2012 to January 2014, 403 people (148 men and 255 women) aged 75 years and above living in the model area of Tome City, Northern Japan, were approached to participate in the study (Akanuma et al., 2016; Liu et al., 2017). Totally, 188 out of the 403 community dwellers aged 75 years and above agreed to participate, and 178 participants out of the 188 completed the WHOQOL-BREF; 66 (32 males, 34 females) scored a CDR of 0 (healthy), 86 (33, 53) scored a CDR of 0.5 (questionable dementia or very mild dementia), and 26 (12, 14) scored a CDR of 1 and above (dementia) (**Table 1**). All patients with a CDR of 1 met the dementia diagnosis using the DSM-IV (American Psychiatric Association, 1994). We utilized the CDR and also conducted clinical examinations, blood tests, neuropsychological tests, and magnetic resonance imaging (MRI). We used the Mini-Mental State Examination (MMSE) as a global intelligence scale (score range: 0–30) (Folstein et al., 1975) and the Barthel Index as a basic activities of daily living (ADL) test (score range: 0–100) (Mahoney and Barthel, 1965). With regard to demographic data, significant differences were found among the three groups in age, educational level, MMSE scores, and the Barthel Index scores using one-way analysis of variance (ANOVA) and the Bonferroni *post hoc* group comparisons, with difference reported as significant if *p* < 0.05. There was no significant difference in gender among the three groups using the two-tailed chi-square test, with differences reported as significant if *p* < 0.05. The participants belonging to the CDR of 0.5 and the CDR of 1 and above groups were significantly older than those of the CDR of 0 group using ANOVA (*F*[2,175] = 9.0, *p* < 0.001) with the Bonferroni *post hoc* test (*p* < 0.05). The educational level of the CDR of 0.5 group was significantly lower than that of the CDR of 0 group using ANOVA (*F*[2,175] = 4.4, *p* < 0.013) with the Bonferroni *post hoc* test (*p* < 0.05). The MMSE scores of the CDR of 0.5 and the CDR of 1 and above groups were significantly lower than those of the CDR of 0 group using ANOVA (*F*[2,175] = 35.2, *p* < 0.001) with the Bonferroni *post hoc* test (*p* < 0.05). The CDR of 1 and above group had significantly poorer Barthel Index scores than did the CDR of 0 and the CDR of 0.5 groups using ANOVA (*F*[2,175] = 36.9, *p* < 0.001) with the Bonferroni *post hoc* test (*p* < 0.05).

**Table 1:** Demographics of participants.

	CDR 0 (healthy)	CDR 0.5 (questionable dementia)	CDR 1+ (dementia)	X^2^/F	*p*-value
N (M/F)	66 (32/34)	86 (33/53)	26 (12/14)	1.7	0.436
Age (y)	79.3 (4.3)	81.0 (3.8)^a^	83.3 (5.2)^a,b^	9	<100.0
Education (y)	10.1 (2.2)	9.2 (1.7)^a^	9.1 (1.7)	4.4	0.013
MMSE	24.7 (3.3)	23.0 (3.0)^a^	17.9 (5.2)^a,b^	35.2	<100.0
Barthel Index	99.2 (3.1)	98.1 (5.1)	86.5 (14.1)^a,b^	36.9	<100.0

*Scores show mean (standard deviation) in age, education, and MMSE. CDR, Clinical Dementia Rating; MMSE, Mini-Mental State Examination. Statistical analyses: sex, χ^2^ test; other variables, ANOVA. Post hoc test (Bonferroni test); ^a^CDR 0, ^b^CDR 0.5, p < 0.05.*

### Assessments

#### Subjective Complaint: WHOQOL-BREF

The WHOQOL-BREF questionnaire is a test used to assess the QOL of healthy subjects and patients with very mild and mild dementia, and it consists of four subscales: physical health, psychological health, social relationships, and the environment domains (The WHOQOL Group, 1998; Saxena et al., 2001; Tazaki and Nakane, 2007). Physical health consists of seven items: ADL, medication, energy, mobility, pain, sleep, and work capacity. Psychological health consists of six items: body image, negative feelings, positive feelings, self-esteem, personal beliefs, and concentration. Social relationships consist of three items: personal relationships, social support, and sexual activity. The environment consists of eight items: finance, security, accessibility of care, leisure, home environment, information, traffic, and transport (The WHOQOL Group, 1998; Saxena et al., 2001; Tazaki and Nakane, 2007). We used the average scores of three to eight items from each domain.

#### Objective Observation: CDR

A clinical team comprised of skilled physicians (three neurologists and a psychiatrist) and skilled public health nurses determined the CDR for each participant (Hughes et al., 1982; Morris, 1993) and were blinded to the cognitive test results. They used the Japanese version of the questionnaire of the CDR scoring sheet (Meguro, 2008). The CDR contains six subscales: memory, orientation, judgment and problem solving, community affairs, home and hobbies, and personal care. Before the interviews with the physicians, the public health nurses visited the participants’ homes to evaluate their daily activities. Observations by family members regarding the participants’ lives were described in a semi-structured questionnaire. Participants who lived alone were visited frequently by public health nurses to evaluate their daily lives. The physicians then interviewed the participants to assess their episodic memory, orientation, and judgment. Finally, with reference to the information provided by the family members, the CDR for each of the participants was determined at a joint meeting of the physicians and public health nurses. One author (KM) was certified as a CDR rater by the Knight Alzheimer’s Disease Research Center’s Memory and Aging Project at the Washington University School of Medicine.

### Analyses

We analyzed the relationships between the six CDR subscales and the four WHOQOL-BREF subscales using Pearson’s correlation coefficient, which was two-tailed, with differences reported as significant if *p* < 0.05. We used the IBM SPSS statistics version 22 software (IBM, Corp., Armonk, NY, United States) for all statistical analyses in this study.

### Ethics Statement

Written informed consent was obtained from each of the participants and their families. The study was approved by the ethical committees of the Tome City government and Tohoku University Graduate School of Medicine.

## Results

**Table 2** shows the relationships between the CDR and WHOQOL-BREF subscales based on Pearson’s correlation coefficient (significance level, *p* < 0.05). The number of subjects was 178 in all statistical analyses. The CDR is a scale of cognitive impairment. Higher CDR subscale scores show more severe cognitive impairment levels, and higher WHOQOL-BREF domain scores show better subjective quality of life levels in the **Table 2**. The CDR subscale of memory impairment had a significant statistical negative correlation with the WHOQOL-BREF subscales of physical and psychological domains; Pearson’s correlation coefficients (r) and the corresponding *p*-values for these correlations were *r* = -0.151 and -0.232, and *p* = 0.044 and 0.002, respectively. The CDR subscale of orientation impairment had a significant statistical negative correlation with the WHOQOL-BREF subscale of physical domain (*r* = -0.166, *p* = 0.027). The CDR subscale of judgment and problem solving impairment had a significant statistical negative correlation with the WHOQOL-BREF subscales of the physical (*r* = -0.171, *p* = 0.023), psychological (*r* = -0.232, *p* = 0.002), and environmental domains (*r* = -0.163, *p* = 0.029). The CDR subscale of community affairs impairment had a significant statistical negative correlation with the WHOQOL-BREF subscales of the physical (*r* = -0.215, *p* = 0.004), psychological (*r* = -0.197, *p* = 0.008), and environmental domains (*r* = -0.157, *p* = 0.036). The CDR subscale of home and hobbies impairment had significant statistical negative correlations with all WHOQOL-BREF subscales including the physical (*r* = -0.226, *p* = 0.002), psychological (*r* = -0.226, *p* = 0.002), social (*r* = -0.167, *p* = 0.026), and environmental domains (*r* = -0.204, *p* = 0.006). The CDR subscale of personal care impairment had a significant statistical negative correlation with the WHOQOL-BREF subscale of the physical domain (*r* = -0.177, *p* = 0.018). Collectively, the physical domain of the WHOQOL-BREF had significant statistical negative correlations with all CDR subscales.

**Table 2:** The relationships between the WHOQOL-BREF subscales and the CDR subscales.

			WHOQOL-BREF subscales			
			Physical domain	Psychological domain	Social domain	Environmental domain
CDR subscales	Memory impairment	*r*	-0.151*	-0.232	-0.072	-0.146
		*p*-value	0.044	0.002	0.337	0.051
	Orientation impairment	*r*	-0.166*	-0.143	-0.059	-0.081
		*p*-value	0.027	0.056	0.433	0.280
	Judgment and problem solving impairment	*r*	-0.171*	-0.232*	-0.140	-0.163*
		*p*-value	0.023	0.002	0.062	0.029
	Community affairs impairment	*r*	-0.215*	-0.197*	-0.107	-0.157*
		*p*-value	0.004	0.008	0.157	0.036
	Home and hobbies impairment	*r*	-0.226*	-0.226*	-0.167*	-0.204*
		*p*-value	0.002	0.002	0.026	0.006
	Personal care impairment	*r*	-0.177*	-0.110	-0.056	-0.089
		*p*-value	0.018	0.145	0.460	0.239

*The number of subjects = 178. Statistical analysis; Pearson’s correlation coefficient, ^∗^p < 0.05. CDR, Clinical Dementia Rating. Higher CDR subscale scores show more severe cognitive impairment levels. Higher WHOQOL-BREF domain scores show better subjective quality of life levels.*

## Discussion

In this study, we investigated the relationships between the subjective measure of QOL (WHOQOL-BREF) and the objective measure of impairment (CDR) among elderly people with/without dementia living in a community. The results of correlation analyses revealed that all CDR subscales significantly correlated with the physical domain of the WHOQOL-BREF. In particular, the CDR subscale of objective memory impairments significantly correlated with poor physical and psychological QOL. Meanwhile, the CDR subscale of home and hobbies significantly correlated with poor scores on all WHOQOL-BREF subscales.

These results can be interpreted in two ways. The first is that Japanese elderly people may confuse objective cognitive impairments with subjective physical health. The second is that the objective home and hobbies impairment affects all categories of QOL in the elderly. Age may be a factor that may affect these correlations. Since our study population was limited to one age cohort aged 75 years or above, we did not choose age as a factor in this study.

**FIGURE 1:**
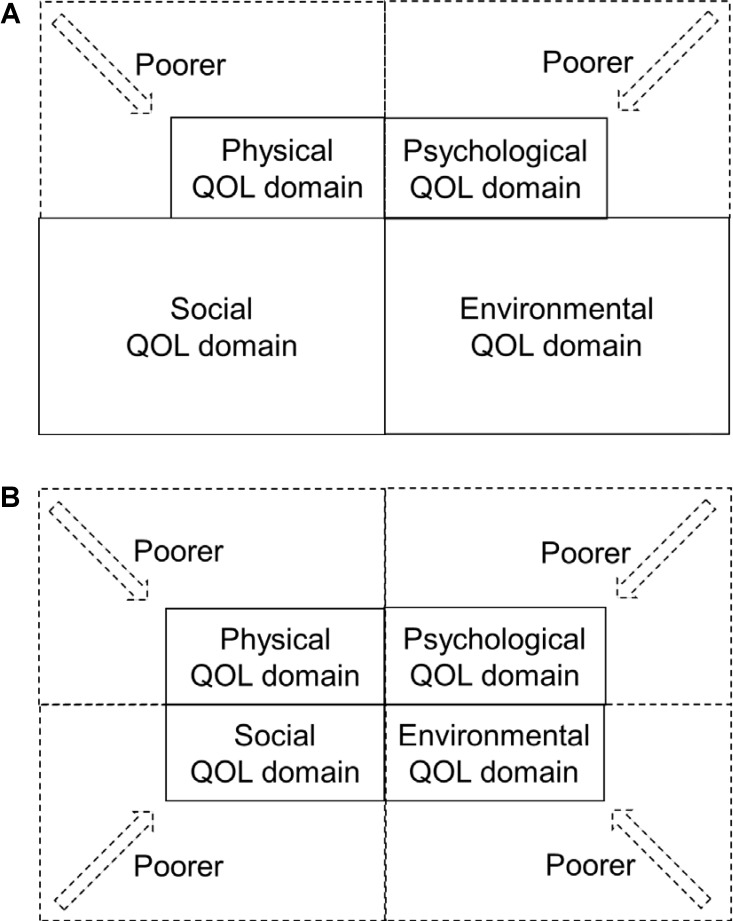
Image of the relationships between objective cognitive impairments and QOL domains. **(A)** Objective memory impairment and each QOL domain. This figure shows the model of objective memory impairment (CDR memory domain) and each QOL domain (WHOQOL-BREF). Objective memory impairment was related to poor physical and psychological QOL domains. **(B)** Objective home and hobbies impairment and each QOL domain. This figure shows the model of objective home and hobbies impairment (CDR home and hobbies domain) and each QOL domain (WHOQOL-BREF). Objective home and hobbies impairment was related to physical, psychological, social, and environmental QOL domains.

First, in our study, the cognitive impairments in all CDR subscales significantly correlated with the physical health domain of the WHOQOL-BREF. As an assessment of “objective cognitive impairments,” we used the CDR scale, which included memory, orientation, judgment, community affairs, home and hobbies, and personal care impairments. Memory impairment, one of the objective cognitive impairments, is the most frequent and severe symptom of dementia, specifically in Alzheimer’s disease (McKhann et al., 1984). **Figure 1A** depicts an image of the relationships between objective memory impairment and the QOL domains. In this study, objective memory impairment correlated not only with subjective poor scores in the psychological domain but also poor scores in the physical domain of the WHOQOL-BREF. When the elderly person actually had “objective cognitive impairments,” he/she may have subjectively had physical complaints such as knee pain or fever. These individuals had shown confusion between objective cognitive impairments and subjective physical health. In our previous study of patients with hip fractures and dementia, the fear of falling—one of the psychological risk factors for falling—reflected not only physical functions but also cognitive impairments (Kasai et al., 2017). It suggested that the fear and concern of falling may reflect not only physical impairments but also cognitive impairments in patients with dementia (Kasai et al., 2017). Another opinion is a potential of cultural difference. For example, elderly Japanese people would view a massage as benefiting both the body and the mind, whereas elderly westerners might view it as benefiting the body only. In this study, there might be some individuals who subscribed to the idea of “mind-body unity,” a term from Japanese Zen Buddhism (Yuasa, 1987). “Subjective physical complaints” related to cognitive impairments may help the early detection of very mild dementia.

Second, when the elderly person actually had “objective home and hobbies impairment,” he/she subjectively felt the decline in all QOL domains. **Figure 1B** shows the image of the relationships between objective home and hobbies impairment and poor QOL domains in Japanese people with very mild and mild dementia. After retirement, most elderly people would be concerned with activities related to their home and hobbies. However, elderly people with very mild dementia exhibited disabilities of complicated IADL activities in the home, such as shopping and food preparation (Kobayashi et al., 2016; Ouchi et al., 2016). Cognitive impairments, especially executive dysfunction, quantitatively and qualitatively decreased the complicated IADL activities in patients with very mild dementia (Ouchi et al., 2016) in spite of the intensity of physical activity (Kobayashi et al., 2016). We need specific rehabilitation of and care for dysfunctions of activities related to home and hobbies among elderly people with very mild dementia through physical, psychological, social, and environmental supports.

An additional hypothesis is that people with very mild or mild dementia may have “anosognosia” or an “over or under estimation of true cognitive impairment” with an imbalance between subjective and objective cognitive complaints, especially in patients with vascular dementia (Tezuka et al., 2013). Elderly people with dementia may misunderstand matters concerning their cognitive and physical impairments. This may be related to the Dunning–Kruger effect. The Dunning–Kruger effect is a term used in connection with an imperfect self-assessment based on the metacognitive deficits (Kruger and Dunning, 1999). People with cognitive impairments may not be aware of their disabilities, therefore self-awareness is required to recognize impairments. Another studies need to investigate the neural basis of the mechanism underlying disabilities of behavior and the subjective and objective awareness of cognitive impairment.

In the future, another studies need to develop and systematically combine memory clinics and all departments related to the elderly, including orthopedics, for the successful early detection and rehabilitation of, and long-term care for, dementia. We also need international comparison studies on people with dementia in terms of their objective and subjective cognitive impairments.

## Author Contributions

MK: data analysis and writing the manuscript. KM: design and direction.

## Conflict of Interest Statement

The authors declare that the research was conducted in the absence of any commercial or financial relationships that could be construed as a potential conflict of interest.
